# Professors of racial medicine: imperialism and race in nineteenth-century United States medical schools

**DOI:** 10.1017/mdh.2024.9

**Published:** 2024-04

**Authors:** Christopher D. E. Willoughby

**Affiliations:** University of Nevada, Las Vegas, Nevada, US

**Keywords:** History of Racial Science, Medical Education, U.S. Slavery, Imperialism

## Abstract

This article examines some of the racist features of nineteenth-century medical school curricula in the United States and the imperial networks necessary to acquire the data and specimens that underpinned this part of medical education, which established hierarchies between human races and their relationship to the natural environment. It shows how, in a world increasingly linked by trade and colonialism, medical schools were founded in the United States and grew as the country developed its own imperial ambitions. Taking advantage of the global reach of empires, a number of medical professors in different states, such as Daniel Drake, Josiah Nott and John Collins Warren, who donated his anatomical collection to Harvard Medical School on his retirement in 1847, began to develop racial theories that naturalised slavery and emerging imperialism as part of their medical teaching.

Like his predecessor and other contemporary anatomists, University of Pennsylvania Anatomy Professor Joseph Leidy (1823–91) discussed racial differences in terms of cranial capacity, brain weight and other alleged anatomical differences in a matter-of-fact way.^1^ In an undated set of lecture notes for his anatomy class, he explained that the ‘smallest skull[s] [were] hindoo’ and ancient Peruvian. He also classified skulls as brachycephalic (short and broad) versus dolichocephalic (long and narrow) and prognathous (projecting jaw) versus orthognathous (nonprojecting jaw). Western Europeans had dolichocephalic orthognathous skulls, he wrote, compared to ‘Negro & Australians’ who had dolichocephalic prognathous skulls, ‘Mongolians’ on the other hand had brachycephalic prognathous skulls. In a lecture on brain weight, he also noted that ‘hindoos’ had some of the lightest brains along with Black people.^2^

As Leidy’s case illustrates, some medical professors in the nineteenth-century United States approached questions of race comparatively and framed them as such for their students, borrowing examples from various colonial territories. This article argues that, as medical schools in the United States began to grow at a rapid pace, the medical study of human remains and data associated with European and American imperial violence increased. Just as scientific understandings of race and slavery enhanced the social power of medical schools, some faculty began to study and comment on race within the expanding United States and abroad. Medical school faculty thus produced and disseminated an essentialist science of race. This science was made possible in part by various imperial networks, and it had implications for diverse nineteenth-century US imperial projects.^3^

The antebellum United States’ imperial ambitions were twofold. On the one hand, the country was expanding into the interior of North America through the conquest of various Native American territories, particularly to the west. On the other hand, the United States was launching missions or creating imperial projects outside its growing borders, for example, with its involvement in Liberia in Africa. Like Liberia’s connections to the American Colonisation Society, many of these projects were tied to a US foreign policy bent towards increasing the United States’s naval power and influence in the Atlantic and Pacific regions. Matthew Karp has shown how proslavery Southern cabinet members advocated for a massive buildup of the US Navy, as an effort to check the British Navy and enforce the Monroe Doctrine, protecting slavery in the Western Hemisphere. Additionally, rather than the formal imperialism of established empires such as Britain or France, these projects just as often included private actors, operating outside official government roles but nonetheless tied to American foreign policy. Given this context, this essay reveals how the imperial ambitions of the United States and the creation of a racial curriculum that complimented those ambitions were relatively simultaneous and mutually informing. It would be an exaggeration to suggest that medical schools directly shaped the foreign policy of the United States. Rather, they disseminated a science of human difference that was ideologically complementary to imperial expansion as part of a racist culture in the United States.^4^

While historians have considered the rise of racial medicine in the United States as primarily, if not almost exclusively, rooted in domestic politics, current research is shedding light on the relationship between imperialism, slavery and the production of racial science in antebellum America.^5^ Scholars of slavery and medicine in the United States have revealed how medical schools in the South used the local enslaved population to create pathological and anatomical collections. Cameron Strang has shown how naturalists along the Gulf of Mexico coast spanning from Florida to East Texas produced scientific knowledge through colonial violence and competing networks of imperial patronage from the British, French, Spanish and later Americans. Other scholars have argued that nascent imperialism in the Caribbean and Pacific regions led to the rise of schools of tropical medicine but mainly from the end of the nineteenth century onwards.^6^ In contrast, scholars of the British and French empires in the nineteenth century have a well-established historiography of how imperialism shaped both medical and racial knowledge production from the early modern period to the twentieth century.^7^

Similarly, as I show in my book *Masters of Health: Racial Science and Slavery in U.S. Medical Schools*, anti-Black racial science was incorporated into medical school curricula in the form of textbooks, lectures, student writing and museum displays. However, I gave little attention to the racial science research being conducted on the stolen remains of Native Americans and other groups mostly encountered through maritime exploration, as this had a much smaller impact on medical pedagogy. That being said, medical faculty conducted racial research about Native Americans and other groups outside of the Black–white binary, even if they discussed these groups infrequently in the classroom. Thus, examining medical faculty’s racial science research on Native Americans, the enslaved and peoples outside of US borders reveals that antebellum medical schools provided a vital hub for the production of increasingly globalised racial science research.^8^ This science complimented the United States’ ambitions toward both continental and global expansionism.

Thus, this article emphasises how, by the mid-nineteenth century, the supposedly scientific concepts of race produced by American medical faculties were shaped by the wider impact of imperial armies and bureaucracies. Although the United States was not an imperial power in the nineteenth century in the same way as the United Kingdom or France, it had imperial ambitions of its own and was linked to imperial networks. Some of the data and specimens used in medical education were collected through the various imperial activities in which the United States was engaged, such as settler colonialism in North America, attempts at overseas expansion, the establishment of the colony of Liberia in Africa and the use of various imperial channels by physicians.

## Contexts in early medical education and imperial aspirations

The rise of the United States’ imperial ambitions and the growth of medical schools share a common timeline and represent two parts of the national development of the country after its independence from the British Empire. The first medical school in the British colonies was founded in Philadelphia in 1765 (now the University of Pennsylvania Medical School), followed by another in New York City in 1767 at King’s College (now Columbia University). Harvard’s medical department was founded in 1783 as the first postcolonial school. In these early years of American medical education, graduation rates were low. Apart from local apprenticeships, physicians in the United States largely looked to Britain and the University of Edinburgh as the centre of Atlantic medical education. In the eighteenth century, medical schools in the United States graduated little more than 200 students. In many ways, these medical institutions developed in much the same way as Martin Robert describes for Canada in this issue. While some schools were founded before the War of Independence with Great Britain, institutional development did not take off until the nineteenth century. The territorial expansion of the United States was also relatively slow during this period, even though conflict with and genocide of indigenous groups had been ongoing in various forms since the beginning of British settler colonialism in North America. It was after the Louisiana Purchase in 1803 that the rapid territorial expansion of the United States under the ideology of manifest destiny began in earnest.^9^ This correlated with the growth of medical schools and the number of doctors in the country.

While white doctors rarely worked on Native American bodies, the expansion of medical schools was directly linked to settler colonialism. The farmland and plantations of the South and the Old Northwest (now the Midwest) had been expropriated from the many Native American societies that had lived in North America for millennia. Westward expansion accelerated in the antebellum period, culminating in the Mexican–American War (1846–1848) and so did the number of medical schools in the South and Midwest. In the 1850s alone, more than 17 000 doctors graduated from medical schools in the United States, which had by then surpassed Canada in the creation of new medical schools and the awarding of medical degrees, compared with a few hundred in the eighteenth century. Many of these doctors would make their living caring for the bodies of the small farmers and enslaved labourers on the farms and plantations of the Midwest, Deep South and, eventually, West. For example, in 1864, a medical school was established for the first time on the Pacific Coast of the United States. American medical education on the Pacific Coast reflected the rapid growth of California’s settler population in the wake of the Gold Rush in 1849 but also a growing interest in commerce and, later, territorial acquisition in the Pacific region.^10^

Plantation agriculture accelerated by the cotton boom in states like Tennessee and was made possible by the dispossession of Native American land, and the enslavement of people of African descent helped create the conditions that led to the collection of human remains for medical education. In 1823, Mary Austin Holley wrote to John Collins Warren, professor of anatomy at the Harvard Medical School, offering to send Warren a Native American’s skull. Originally from Connecticut, Holley knew Warren from having lived in Boston with her husband Reverend Horace Holley in the 1810s. Horace Holley was the president of Transylvania University when they travelled around the Old Southwest and stayed on a plantation with a large Native American burial mound. The elderly plantation owner, Reverend Craighorn, had already partially exhumed the mound, finding many skulls in good condition. ‘Not only skulls, but complete skeletons, may be found in the mound’, Holley asserted. Understanding that disturbing these remains was against the wishes and beliefs of the tomb’s inhabitants, Holley even joked that, ‘It was well for us that the spirits which once animated these bones did not rise with them. They would have called us *heathen* & worse than *barbarians*’. Craighorn was willing to part with some of the remains found on his slave plantation. All Warren had to do to acquire them was to hire a local agent to pack and ship them to Boston via New Orleans. Alternatively, Holley explained in a postscript, ‘The poor old gentleman is almost blind, through a cataract, & would give all his skulls & everything he has to boot if you could operate on his eyes’.^11^

As the United States expanded its territory in North America, its medical schools matured in the context of overseas expansion and the buildup of the US Navy. The expansion of slavery was a major factor in many of the invasions in which the United States or its citizens were involved during this period. Southern enslavers regularly led the US War, State and Naval departments. This trend gave a distinctly proslavery influence to the foreign policy of the United States. In the 1840s, the State Department used the Monroe Doctrine to try to dissuade Britain from annexing Cuba and abolishing slavery there. President John Tyler even reportedly promised to take naval action against Britain without congressional approval if it attempted to take Cuba. A decade later, the proslavery imperialists went further when Southerners supported private ‘filibuster’ wars in Cuba and Nicaragua. As secretary of the Navy, another Southern politician, James Dobbin, pushed for and supported the United States’ armed trading expedition to Japan led by Commodore Matthew Perry in 1854. This military venture was intended to signal the United States’ entry onto the world stage. Dobbin also sought naval influence in the Pacific region, overseeing a failed expedition to Panama in the hope of building a canal that would make the Caribbean a trade route between the Pacific and Atlantic region. By projecting the nation’s burgeoning naval power in the Greater Caribbean and Pacific regions, American expansionists hoped in particular to escalate the ongoing maritime competition with Britain.^12^

In the United States, some of these expeditions had an impact on racial science and science in general. From 1838 to 1842, a group of scientists, sailors and explorers circumnavigated the globe on the US Exploring Expedition. This voyage laid the foundation for the establishment of the Smithsonian Institution. Expeditioners even abducted a Fijian man named Veidovi, who died shortly after his arrival in the United States and whose skull was ultimately incorporated into the museum’s physical anthropology collections. Likewise, the multivolume publication of the expedition’s reports was one of the Smithsonian’s first major undertakings. As part of these reports, Harvard medical graduate and physician Charles Pickering produced a racial monograph based on his experiences with the expedition, entitled *The Races of Man; and their Geographical Distribution* (1850). Based on his travels, Pickering made a lengthy argument in favour of polygenesis – the theory that each race was created as a separate species, with different anatomy and susceptibility to diseases and climates. As evidence of the book’s influence on medical education, Harvard Professor of Anatomy Oliver Wendell Holmes Sr. commissioned enlarged reproductions of images from Pickering’s monograph for use in his annual lectures on race, given as part of his larger anatomy course.^13^

Pickering was among many racial scientists who sought to compare American populations to those met by American explorers, naval forces and scientists. For example, during an 1857 expedition to the Dutch colony of Suriname, Harvard Professor of Comparative Anatomy Jeffries Wyman revealed in his diary entries how he racially analysed the foreign population. He noted, for example, that the local Charib Indians had not been ‘degraded’ by their contact with civilisation, unlike other groups.^14^ While expeditions like Wyman’s were less common, other doctors from the United States travelled abroad during this period through service on naval and merchant ships.^15^

## Racial science research among medical faculty

Simultaneously, the United States began to take steps to become an imperial power in its own right. In 1820, the American Colonization Society began sending formerly enslaved and other free Black people to the pepper coast of Africa, which later became the country of Liberia. The Colonization Society and the federal government hoped that the freed people would create a colony for the United States, where they could send more formerly enslaved people. The colonisers claimed to want to end slavery, but their central aim was to maintain white dominance in North America.^16^ Indeed, colonisation had a specifically polygenist logic: returning Black people to the continent and environment for which they were supposedly created.

As Robert Murray points out, many early Black graduates of United States medical schools were admitted in relation to the colonisation of Liberia. The first Black medical graduate in the United States, Samuel F. McGill, was a Black Liberian settler born in the United States. Likewise, at least two of the first three Black students admitted to Harvard’s medical school in 1850–1851, while they were never allowed to graduate, were required to agree to emigrate to Liberia. However, white student protests quickly ended the short-lived integration, with the students possibly not completing even a year of study.^17^

Public advocacy for colonisation revealed how the debate over Southern slavery and environmental concepts of race and health had implications beyond the borders of the United States. In a series of letters from Daniel Drake, professor of pathology and the practices of medicine at the University of Louisville, to John Collins Warren in 1851, later published in the influential *National Intelligencer*, Drake captured some of the imperial dimensions of antebellum racial science. By this point, Drake had long been a professor at the University of Louisville Medical School and the Medical College of Ohio. Introducing the series of letters, the paper’s editors described Drake as ‘one of the most eminent citizens of the Western country’.^18^ In the last letter, Drake displayed how support for the concept of race and support for slavery were not inextricably linked. Drake was not an abolitionist and opposed the movement, but neither was he a political supporter of slavery. While Drake defended slave owners, his politics were colonisationist, hoping to end slavery by encouraging Black people to emigrate to Liberia. Most critical for Drake was the potential consequences of Black people being freed from enslavement and entering a northern climate that was supposedly destructive to their health. Thus, Drake reinforced biodeterminist beliefs about race and the environment, even as he supported monogenesis. He believed constitutions could change, unlike polygenists. These ‘changes of constitution’, however, ‘are slowly made’, Drake explained, ‘and the colored people of those [southern] States still inherit a capacity for enduring heat, but not of bearing cold. Thus, in the cities of the South, they seldom suffer yellow fever; and the Virginia planter who removes to Florida is much more liable to its fevers than his slaves’. In Liberia on the other hand, Drake asserted, ‘the fevers which prevail are of the very same kind with those which occur every year in Mississippi and Alabama’.^19^ Claiming that the southern United States and Liberia shared diseases and climate, he concluded that the only safe place for people of African descent was Africa. Drake even called for the northern states to ban the entry of newly free Black people from the South. Thus, while not in complete agreement with the polygenists, much of the practical prescriptions and underlying medical logic were shared.

Beyond popular articles like Drake’s, United States racial scientists and physicians used European imperial data on health and race in the tropics to make arguments about the health-related outcomes of overseas imperialism, which they disseminated through medical journals, bureaucratic reports and books. These data were transmitted through a global network of connections created by imperialism and the maritime trade that it generated.^20^ As historian Jim Downs explains, ‘[a] bureaucracy that had been established in service of war, colonialism, and imperialism emerged as the foundation for the development of epidemiology’.^21^ The data produced by these bureaucracies were often ideologically bent. The addition of racial demographic data encouraged racial scientists to repurpose this epidemiological research for the study of race. Even in the present, epidemiological data using racial categories can still, without proper contextualisation, have the effect of making race appear as a biomedical rather than a social category.^22^

Data collected in the nineteenth century often illustrated this dynamic. It lacked the context of the social and material conditions of those studied, and racial categories were given the appearance of explaining racial differences in health. But their interpretation and use were far from consistent. Drake and other American monogenists of the period, such as John Bachman, represented a sort of middle ground between earlier generations of monogenists, who believed that racial differences were fleeting, and antebellum polygenists, who saw them as permanent. For the fate of many nonwhite people around the world, however, these distinctions were academic.^23^ As a result, more critical than these academic disagreements was the broad consensus that emerged around notions of racial difference, health and the environment.^24^

In his 1857 essay ‘Acclimation; or, the Comparative Influence of Climate, Endemic and Epidemic Diseases, on the Races of Men’, surgeon and anthropologist Josiah Nott grafted polygenesis onto the politics of race, health and imperialism. This essay was a part of Nott and his collaborator George R. Gliddon’s second book, *Indigenous Races of the Earth* (1857). By this time, Nott was professor of anatomy at the University of Louisiana (now Tulane University) and later founded the Medical College of Alabama. Having trained at the University of Pennsylvania and in the clinics of Paris, he was a widely respected figure in American and transatlantic medicine. Thus, Nott’s influence was widespread in both popular culture and the medical community, including through his students. His propolygenesis books, written and edited with George R. Gliddon, included contributions from Ivy League faculty such as Joseph Leidy and Louis Agassiz. In addition, anatomy faculty such as Leidy and Harvard’s Oliver Wendell Holmes clearly reflected the influence of Nott and other polygenists in teaching their students about alleged racial differences.^25^

In his essay, Nott questioned the medical wisdom of imperial expansion for whites and nonwhites alike. In particular, he argued that each race was designed for a specific medical climate, with geographically ‘prescribed salubrious limits’. Whites could survive in the tropics and the tundra, but their life span would significantly decline if they lived outside of a temperate climate. People of African descent achieved optimum health in the tropics but could survive in temperate zones. To demonstrate the comparative and geographically wide scope of this work, Nott included colonial data on the labouring classes of Egypt and Algeria. Domestically, United States physicians like Nott framed the South as an ideal space for plantation slavery, suited to the occupational health of the white planter and the Black slave. The same dynamic applied to multiracial colonial spaces such as parts of India. ‘Not only in these more temperate regions of the United States is this proximity of the two climates observed’, Nott explained, ‘but also in Bengal and other parts of India, in the islands of the Indian Ocean, at Cape Colony, the West India islands, &c’. Domestically minded and detesting racial mixing, Nott advocated medical and political isolationism. But in his research and reading tastes, Nott was anything but an isolationist. His arguments about the health of Black people in the South were closely linked to his understanding of the relationship between British and French imperial agents and colonised peoples in South Asia, Africa and elsewhere. He saw his science as relevant beyond national borders, even as he targeted the domestic politics of the United States.^26^

Nott’s ideas on race had potential for wide application, reflecting the emergence of a nascent global dataset on race, health and the environment. Physicians like Nott could use this data to contextualise local racial differences within larger trends. As an example, Nott discussed a British colonial force of white and Black West Indian soldiers serving in Gambia. While nearly all of the 300 white soldiers died, the Black regiment lost only one soldier, according to Nott’s sources. He explained, ‘these black soldiers, too, had been born and brought up in the West Indies; and according to the commonly received theory of acclimation, should not have enjoyed this exemption. No length of residence acclimates the whites in Africa’. Earlier, he explained that there was ‘no reason to believe the Anglo-Saxon can ever be transformed into a Hindoo’. To support his broader claims, Nott provided mortality data from Algeria, Batavia, Trinidad, Sainte Lucie, Martinique, Guadeloupe, Bombay, Calcutta, Havana and Granada. He even gave separate data for certain locations based on race, caste and occupation. Nott’s work did little to bolster the supposed impartiality of statistical data. Instead, it highlighted how both quantitative and qualitative data were filtered through the racial assumptions typical of antebellum white culture. He also revealed how European imperialism informed racism in the United States and its development in medical education.^27^

Nott’s beliefs were influential. Harvard Anatomy Professor Oliver Wendell Holmes Sr., Medical College of South Carolina Anatomy Professor J. Edwards Holbrook and University of Pennsylvania Anatomy Professor Joseph Leidy all were influenced by Nott’s views on race.^28^ More substantively, in an 1856 letter to Nott, Professor of the Institutes and Practices of Medicine Samuel Henry Dickson also asserted that Black people were suited for toil in the tropics and subtropics, marshalling a geographically wide array of anecdotal data. By this time, Dickson had been teaching at the Medical College of South Carolina for more than thirty years, apart from a brief stint at New York University Medical College. He would finish his career teaching at Jefferson Medical College in Philadelphia from 1858 to 1874. Dickson explained, ‘The Anglo-Saxon race can never become acclimated against the impression of intermittent and bilious fever, “periodical,” or “malarious fevers”’. In support of such an assertion, Dickson like Nott cited French medical geographer Jean Christian Marc Boudin, explaining that Boudin:

argues against the possibility of such acclimation, dwelling upon the little success of and great mortality attending the colonisation of Algeria, the European and English intrusion in Egypt and into Hindostan. The French, he tells us, cannot keep up their number in Corsica. In the West Indies, the white soldier is twice as likely to die as the black; in Sierra Leone, sixteen times more likely; and this continues permanently.^29^

Drake, Dickson and Nott captured how ideas about race and climate held by many United States medical faculty lay on a spectrum. Their ideas were neither universal nor consistent, but in practice they all argued that Black people were unhealthy in the North and in cool climates. Increasingly, they also applied this frame to diverse groups outside of the United States.

Occasionally, some antebellum medical students also discussed climate, race and health in transnational and comparative frameworks in their senior theses. During this period, theses tended to reflect medical pedagogy rather than original analysis, making them valuable sources for the study of medical school curricula. Among the more than 4 000 extant antebellum student theses from the Medical College of South Carolina and the University of Pennsylvania, 10 percent of students included discussion of racial difference in their theses. A small subset even applied racial theories to populations outside the United States. For example, in 1839, David M. Watson, a student at the Medical College of South Carolina in Charleston, noted that Black people and ‘Hindoos’ were prone to scrofula. Five years later, another student at the Medical College of South Carolina, Isaac Auld, stated that, ‘Negroes and Hindoos are unusually prone to Scrofula when they come to temperate climates’. Auld was particularly detailed here. He compared races and considered migration across climates. In discussing diseases such as tetanus, dysentery and malaria, to name but a few, students from the University of Pennsylvania and the Medical College of South Carolina invoked authorities and accounts of race and health from the West Indies. Another student made claims about the comparative birth rates of Egyptians and ‘Africans’ in Egypt, claiming that Africans (presumably Black people) had higher fertility rates. He also claimed that this trend in birth rates could be caused by lifestyle.^30^

More common than specific claims were the imaginative racialisation of different peoples and climates by medical students. In his 1837 thesis, Andrew McBryde, a student at the Medical College of South Carolina, captured this evolving comparative racial imaginary and its relationship to medicine, writing: ‘It seems to be a fact that each race of men was designed by natural conformation to occupy a particular climate, etc. suited to the constitution of that race, and adapted to promote the health and well-being of its possession’. Giving a specific example, he continued, ‘The African is withered before the blasts of Greenland’.^31^

American racial theorists were hardly defining American foreign policy and its imperial aspirations, but politicians and important bureaucrats in charge of American foreign policy were clearly paying attention to this new science. Many influential public officials subscribed to either one or both of Nott and Gliddon’s major texts *Types of Mankind* (1854) and *Indigenous Races of the Earth* (1857). For *Types of Mankind*, Secretary of State Edward Everett, Secretary of the Navy John P. Kennedy and Secretary of the Interior A. H. H. Stuart obtained copies for their respective departments’ libraries. Everett and Kennedy also personally subscribed. Other operatives in American foreign policy subscribed to one or both of the monographs like John M. Daniel, envoy to Turin and editor of the *Richmond Examiner*, Commander of the East India Squadron Commodore F. A. Parker, Capt. Charles Wilkes who commanded the US Exploring Expedition and former Secretary of State and Treasury Louis McLane. Senators including Charles Sumner (Massachusetts), James H. Bayard (Delaware), James Henry Hammond (South Carolina) and Robert Toombs (Georgia) also subscribed. Hammond and Toombs in particular were vociferous supporters of an aggressive, expansionist foreign policy.^32^

Hammond even echoed racial theories of health and climate to justify this policy. In an 1853 essay in *The Southern Quarterly Review*, South Carolina Senator James Henry Hammond made a protracted argument for the United States and Brazil to increase trade relations. The two countries, Hammond believed, shared a climate and an economic foundation in slave labour. In addition to open trade, Hammond argued, Brazil should welcome Southern planters and their enslaved labourers to emigrate, further solidifying hemispheric ties. Here, medical notions about climate, race and health shaped Hammond’s policy. White men could not develop lower Amazonia, Hammond explained, by their ‘unaided manual exertions. The rays of a vertical sun are too intense to be borne by’ them. The answer was African labour. Even better for Hammond, Brazil maintained an active, if illegal, transatlantic slave trade, making this relationship even more enticing.^33^

Following this internalist proslavery agenda, Hammond continued that, ultimately, Southern slaveowners would have to expand the borders of the slavocracy, in the face of decades of compromise that confined the institution to the US South. Eventually, slaveholding Southerners would have to expand beyond these border as the enslaved population grew. Nearing the crescendo of his argument, Hammond asserted, ‘When the necessity arises, the South will *break* the cordon established by the governments; she will incorporate one after another, the Mexican States … California, and even Oregon’. Hammond followed this up by musing on the benefits of annexing Cuba and potentially reopening the slave trade, as he rested his case. He concluded with the ominous sentence, ‘There are many ways of *carrying the war into Africa!*’ In short, Hammond used theories about race, health and the environment to advocate for an expansionist foreign policy that included reopening the slave trade, free trade and open immigration policies with other slaveholding nations in the hemisphere and overt territorial expansion in North America and the Caribbean.^34^

In many ways, unlike Hammond’s political musings, Nott’s data-driven essay was on the cutting edge of racial science in the United States in 1857. Still, these theories were significant. They revealed the nascent influence of an imperial approach to racial medicine and environmentalism well before the rise of tropical medicine in the United States. Likewise, politicians used this discourse to support a proslavery expansionism.

## Imperialism and the curation of racial anatomy in medical school museums

Discussions of race, empire and climate in medical education in North America were also reflected in the collection of skulls and other anatomical specimens. Going back to the eighteenth century, many anatomists and physicians had pioneered some of the methods of racial science and proto-anthropology that became popular in the nineteenth century. For example, Petrus Camper, an eighteenth-century Dutch physician and graduate of the University of Leiden, first measured facial angles to describe racial types. For the same purpose, Samuel Morton, the trailblazing American craniologist and skull collector, housed his vast collection of skulls in the Museum of the Academy of Natural Sciences of Philadelphia. The history of early skull collections has often been framed as the history of early anthropology rather than the history of medicine. In doing so, historians have created an artificial division based on the later development of anthropology as a separate discipline during the professionalisation of science at the turn of the twentieth century. This partly explains why the history of racial science in nineteenth-century American medicine has been less studied until recently.^35^

By the 1850s, and even earlier, medical school museums commonly had racialised skull collections. In the case of skull collecting, medical schools such as Harvard, the University of Pennsylvania and the Medical College of Ohio became complicit in the violence of European empires by collecting racialised human remains. European colonial wars, diplomatic relations and wars of expansion into Native American territories facilitated the collection of these remains in the South and Midwest, as well as in the urban centres of the Northeast.^36^ Moreover, as maritime trade and empire shrank the world, the mobility of human bodies gave racialists in medical faculties access to increasing supposed evidence about race. These remains were then used to create arguments about racial difference, health, and civilisation that supported the logic of imperial expansion.^37^

For example, until he donated it to Harvard in 1847, Professor John Collins Warren had his own private anatomical collection, to which Harvard students had access for much of Warren’s tenure. Warren likely saw the national skulls as the centrepiece of Harvard’s anatomical museum. In the earliest catalogue of the museum, handwritten by Warren and presented to Harvard in 1847, the skulls were the first objects listed. Among thousands of more pedestrian anatomical and pathological objects, Warren donated a total of fifty-six skulls and casts of human skulls, including thirteen ‘Caucasian’ heads, thirteen heads of the ‘Mongolian or Tartar race’, ten ‘Peruvian’ heads, twelve Native ‘American’ heads, and eight ‘African’ heads. These categories also reflected political and moral questions related to American imperial ambitions. For both ‘Mongolian’ and ‘African’ skulls, these represented groups that American ‘Caucasians’ hoped to either enslave or conquer. In contrast, Warren saw superior and ancient ‘Peruvian’ skulls as proving that Native ‘Americans’ had conquered and displaced groups like the Incans who had built the architecturally great civilisations of American antiquity. Moreover, according to Warren, that supposedly inferior Native Americans had displaced the civilised Peruvians justified whites displacing contemporary Native Americans. Simultaneous to donating his collection, Warren also acquired the collection of the defunct Boston Phrenological Society. Professor of Surgery J. B. S. Jackson was by this time curator of both the newly established Warren Museum and the collection of the Boston Society for Medical Improvement, which Harvard would later formally acquire. As a result, by 1847 Harvard students had access to more than 150 human heads, some casts and some originals, arranged by race and nation.^38^

Harvard’s skull collection, like others, was underwritten by the growing naval power of the United States. In 1855, Dr. Jenks H. Otis, a naval surgeon, donated a skull to Harvard’s medical school. According to J. B. S. Jackson, the museum’s curator, the skull was ‘wanting’ in the ‘jaw + teeth’ and the cranial capacity was 89 inches. There was little to distinguish this skull. It had neither an abnormally large nor small cranial capacity compared to others in the collection. Where this skull stood out was in its provenance. Otis had been a surgeon on Commodore Matthew Perry’s armed trading expedition to Japan in 1854. He took the opportunity to steal or buy a skull found on the island of ‘Lew-Chew’ or Okinawa. Harvard, in turn, received a new skull to add to its growing collection.^39^

Medical schools also profited from the expanding skull collecting within North America. Medical schools and individuals such as Philadelphia racial scientist and Pennsylvania Medical College Anatomy Professor Samuel G. Morton created racial skull collections by stealing and purchasing human remains. Anatomy museum curators also procured trophy heads from victims of imperial wars with Native Americans. In 1841, during the Second Seminole War (1835–1842), Dr. Joseph Walker donated the ‘head of Powhushajo, a Seminole Warrior’ to the Anatomical Museum of the Medical Department of the University of Pennsylvania.^40^

Furthermore, skull collectors and medical professors relied upon the networks created by European empires and diplomats. For example, through the intermediary of the historian and Bostonian William H. Prescott, Spanish envoy to Mexico Ángel Calderón de la Barca y Belgrano gave John Collins Warren two skulls from ‘Othonie [?] Indians’ in Mexico. This was meant as a consolation. Calderón had tried in ‘vain to get a genuine skull of the ancient Aztec’.^41^ In a similar fashion, the race scientist and diplomat George Gliddon sent Samuel Morton more than a hundred skulls from his post as vice consul of the United States in Egypt, with origins ranging from East Africa to South Asia. These diplomats acted as conduits for collectors, and physicians benefited from the rapid increase in the quantity of human remains available. Bodies were also easily transported, and collectors relied on the geographically wide dispersal of educated whites in diplomatic posts like Gliddon to find bodies in regions where few American professors ventured.^42^

Skulls were traded as part of a larger culture of taking ‘trophies’ during imperial warfare. Compared with their European counterparts; however, the medical schools of the United States were not able to rely so heavily on their own armies or colonies for specimens. Harvard possessed at least two specimens from the British colony of South Africa, even though there were no American troops stationed there. The first was the skeleton and death cast of a KhoiKhoi man who committed suicide in Boston in 1861. The young man had been performing in an P. T. Barnum exhibit along with four other men from southern Africa, where each represented distinct ethnic groups. The imperial connections were transparent in this case. According to local newspapers, the curators of the exhibition were ‘under bonds to the English Colonial Government in South Africa’. The other was the hair of a female ‘bush man’ who had been in a living display at the age of nineteen in 1862. Other examples include the head of an Inca child, stolen from an abandoned Inca Temple of the Sun and donated to Harvard’s museum by Dr. H. A. Ward. Harvard also owned the skull of a Chinese opium smuggler beheaded by the Qing Dynasty during the first opium war with Britain and displayed on the Pearl River in southern China. Thus, some Harvard professors used their extended reach to turn the Warren Museum into a home for human remains taken from battlefields, stolen from cemeteries and torn from sacred sites.^43^ Despite the slow expansion of the United States outside of North America, physicians and skull collectors maintained a trading power that spanned much of the world and shaped part of their teaching about race.^44^

By arranging the skulls according to their understanding of race, the Harvard medical professors racially linked people whose lives had been widely separated by time, space and circumstances, claiming that they were racially the same, as when Jackson displayed the skull of an ancient Egyptian a shelf away from that of a nineteenth-century Austrian. These were grouped together as white. Beyond the mere metric of size, what made collections like Harvard’s significant was their intent: to educate physicians in a global racial hierarchy.^45^

One case in particular shows how racialised human remains came to Harvard through deeply complex circumstances that reflected the imperial networks and increasingly rapid transportation of the nineteenth century. In 1860, Harvard University’s Warren Anatomical Museum used its funds to purchase the remains of a Native American man. The remains were of a ‘flat-head’ man whose people lived in and around the Columbia River in the Washington Territory. Previously part of the Oregon Territory, the United States had claimed this vast region just over a decade earlier.^46^ Like some other Native American groups, various peoples along the Columbia River compressed and manipulated their skulls as adolescents, creating the effect that Harvard scientists called a ‘flat-head’. Racial scientists, such as the museum’s late namesake and founder John Collins Warren, had fetishised these skulls, seeing them as linked to Inca skulls that had undergone similar manipulation.^47^

But his case was not typical, because while this man grew up in what the whites called ‘Washington Territory’, he died in Boston.^48^ Moreover, his body was not removed from a local burial ground in the Pacific Northwest of the United States, as would have been more typical of remains displayed from that community. Instead, missionaries had persuaded the unnamed man to travel to Europe to further his education. He died of tuberculosis in Boston in 1860, aged just 22. The curator of the Warren Museum paid a local undertaker seven dollars for the body. He paid another ten dollars to a local craftsman, Pietro Garibaldi, who made a cast of the man’s head. Then, following standard procedures for preserving bones and turning them into museum objects, the faculty, including comparative anatomist, racial scientist and physician Jeffries Wyman, used acid or boiling water to remove the flesh and transformed his body into a skeleton. The Harvard faculty then turned the body into several museum objects. Among other body parts, Harvard preserved a death cast of the man’s head, his skull and the skin of his pubic area, with the hair intact. The museum’s curator, Professor of Surgery J. B. S. Jackson, probably acquired the man’s remains because of his ‘flat-head’. Harvard had many other skulls of ‘flat-head’ Native Americans from Peru, the Pacific Northwest and other parts of the Americas.

Professor J. B. S. Jackson, the museum’s curator, took measurements to define the man’s race. While examining the humeri, he noticed a large opening in the olecranon fossa, a part of the humerus near the elbow joint. According to museum records, Dr. C.T. Jackson had observed ‘many years ago’ that this was a common feature among Native Americans. Harvard Professor of Comparative Anatomy Jeffries Wyman had also noted that this was a common trait ‘in about 50% in the Negro race’. This enlarged opening reinforced the professors’ beliefs that Native Americans possessed a distinct anatomy. More critically, given that they had acquired the remains on the basis of the man’s racial categorisation, the faculty saw an opportunity to draw similarities between the supposedly inferior groups in the United States: Native Americans and Black Americans. In the published description of the death cast of the man’s head, Jackson also gave measurements from ‘Vertex to soles of feet’, the breadth of the head of the humerus, leg length, shoulder width, foot length and hand length. He concluded with a racial description of the toes, saying that they ‘overlapped about as much as in Caucasians generally; but there were no corns’.^49^

Yet, collections of human remains with imperial sources were not only held at elite, northeastern institutions such as Harvard or the University of Pennsylvania. Many students in the South, Midwest and West had access to such collections. In the 1850s, George Gliddon donated two mummies from ancient Egypt to the University of Louisiana. In San Francisco, the Pacific Museum of Anatomy and Natural Science had five Egyptian mummies ‘brought from the East by Captain Grant’. Mummies were seen as critical evidence for the antiquity of racial differences. In addition, in their ‘Pathological Room, *For reference and use of Medical Gentlemen and Students only*,’ they possessed the ‘head of a negro; [displaying] the effects of syphilis of a peculiarly virulent character, on the African Coast’ and the ‘skull of a native of Alaska’.^50^

The skull collection of Professor Reuben Mussey is an even clearer example of how the integration of medical schools into imperial networks extended beyond the largest and oldest schools located in the northeastern United States. In 1838, Reuben Mussey was professor of surgery at the Medical College of Ohio. He was also professor of anatomy and surgery at Dartmouth College. Teaching at two medical schools was not uncommon and in some cases financially imperative. Every year for nearly two decades, Mussey travelled from New England to the Midwest to fulfil his teaching obligations. When Mussey travelled, he seemed to carry thousands of anatomical specimens, which he described in a catalogue for his Ohio students. Mussey’s cabinet contained everything a student could want to understand gross anatomy, from the wired skeleton of a hand to the valves of a human heart. Another feature of Mussey’s cabinet was his pathological collections, which included hundreds of specimens. Among them, Mussey had a collection of fifteen human skulls, arranged by ethnicity, along with two others included in this collection as ‘deformed’ but with no ethnic or racial background listed.^51^ With fifteen racial skulls, Mussey’s collection was far less extensive than that available to students at Harvard or the University of Pennsylvania. But for his students in Ohio, Mussey brought evidence used to validate racial difference and craniometry. Much more than racial violence in North America, Mussey’s collection was built on violence in South Asia, Oceania and South America. It seemed particularly indebted to British imperialism. The very first skull was that of a ‘Madras Sapoy’, probably a native of Madras who served in the infantry of the British East India Company. He also had the skull of a Malay, three from Bengal, another of a Bugese, one from Java, two from China, one from New Zealand and two from Surinam. The violence of the British – a function of their expanding influence in the Indian and Pacific regions – was at the heart of Mussey’s collection. Mussey also had the skulls of two North American Indians, one from New Hampshire and another from Illinois. In the collection labelled ‘human crania’, there were two others that were not clearly racialised. One was ‘deformed’, and the other had ‘great occipital development’. Despite being in a designated human crania section, this unit was not the totality of Mussey’s crania. He had eight other specimens of crania and faces in the pathological collections and four others for gross anatomy. None of these were racialised or described in terms of their national origin and were therefore presumed to be white. In short, the collection labelled ‘human crania’ contained only deformed and nonwhite skulls, implying an inherent similarity between the two labels.^52^ Human remains were not the only items that evidenced Mussey’s reliance on a geographically wide array of collectors. He had hundreds of sets of animal bones, including ‘an entire skeleton of an ourang outang’ and the ‘fossil rib of a megatherium’, an extinct giant sloth native to South America.^53^

What made Mussey’s collection most interesting was not its overwhelming size, its uniqueness or the depth of his knowledge of these objects. In each of these respects, Mussey’s collection fell short of similar collections at the University of Pennsylvania or Harvard. Rather, it revealed the subtle ways in which imperial violence influenced medical education throughout the United States. Even students far from the major coastal cities could measure and hold the bones of people from slave colonies such as Suriname or of a Madras sepoy.

## Conclusion

This essay has shown that the history of research and teaching on racial science in medical education in the United States was hardly confined to the borders of the country, or even of North America. Instead, it was through the violence of slavery and settler colonialism on the American continent but also through the broader forces of European and nascent American imperial expansion worldwide that medical school faculty created their racial theories. Specifically, faculty used world-spanning empires and commercial networks to collect skulls and other anatomical specimens, as well as data created by European imperial physicians to interpret race and mortality in different parts of the world. As the United States was not a world empire at this time, they relied on trade with larger imperial states such as Britain and France. The ideologies reinforced by such exchanges, through medical professors such as Josiah Nott and Joseph Leidy, placed the racial system of the United States in a comparative, global context. In short, by paying greater attention to imperial connections, scholars can begin to unpack the trade in ideas and human remains that shaped notions of racial hierarchy in the nineteenth-century medical world and beyond. This approach was inherent in the comparative framework of polygenesis adopted by many physicians at the time.

Therefore, as medical schools and academics in the United States continue to reckon with their institutions’ historical entanglement with transatlantic slavery and the genocide of Native Americans, scholars must avoid the trap of viewing these issues solely through the confines of national politics and borders. Assuming a national framework means that the connections between medical education and racism cannot be completely understood. It forecloses opportunities to analyse the wider aspects of the history of exploitation by medical professionals.^54^ As this article has shown, racial science has been largely constructed through empires on a global scale, both practically and intellectually.

## Footnotes

1

William E. Horner , A Treatise on Special and General Anatomy, 2nd edn., two volumes (Philadelphia: Carey & Lea, 1830), 1: 208–16; Joseph Leidy , An Elementary Treatise on Human Anatomy (Philadelphia: J. B. Lippincott, 1861), 65–92 , 514, 588, 595, 634, 636–37. T. G. Richardson , Elements of Human Anatomy: General, Descriptive, and Practical (Philadelphia: Lippincott, Grambo, 1854), 165–68. For more on textbooks and racial science, see Christopher D. E. Willoughby , Masters of Health: Racial Science and Slavery in U.S. Medical Schools (Chapel Hill: University of North Carolina Press, 2022), 96–100 .

2 Joseph Leidy, Envelopes 8 and 10, Notes to his Course on Anatomy at the University of Pennsylvania, undated, Medical Heritage Library, The College of Physicians of Philadelphia, Philadelphia, Pa. Leidy wrote out his notes on scrap paper. Some scraps had dates with clues as to when they were given, but his racial lectures did not, although the lectures were rewritten and repeated over time, there were only slight variations. For more on Leidy’s racial pedagogy prior to 1861, see Willoughby, *op. cit.* (note 1), especially chapter 4.

3 It should be noted that the medical profession was in a particularly vulnerable position in the middle of the nineteenth century. The profession experienced serious challenges from alternative healers. Moreover, it would be misleading to assume that the medical profession was the major cultural force that it would become in the age of eugenics and afterwards. That being said, it is important to recognise a nascent but growing medical influence on popular ideas of racial difference. In term of the politics of slavery, this is already widely recognised but less attention has been given to groups outside of the United States. For more on race, medicine and slavery, see Rana Hogarth , Medicalizing Blackness: Making Racial Difference in the Atlantic World, 1789–1840 (Chapel Hill: University of North Carolina Press, 2017) and Jenifer L. Barclay , The Mark of Slavery: Disability, Race, and Gender in Antebellum America (Urbana: University of Illinois Press, 2021).

4

Brian Rouleau , With Sails Whitening Every Sea: Mariners and the Making of an American Maritime Empire (Ithaca: Cornell University Press, 2014), 5. Matthew Karp , This Vast Southern Empire: Slaveholders at the Helm of American Foreign Policy (Cambridge: Harvard University Press, 2016).

5

Sven Beckert , Empire of Cotton: A Global History (New York: Alfred A. Knopf, 2014) and Zach Sell , Trouble of the World: Slavery and Empire in the Age of Capital (Chapel Hill: University of North Carolina Press, 2021).

6 Scholars of race and skull collecting in the United States have certainly acknowledged the international nature of these collections, but the implications of the collections for antebellum racial theorising remain less analysed. In his fantastic monograph on the late nineteenth and first half of the twentieth century, Samuel Redman, for example, certainly describes the ethical concerns, salvage anthropology and collecting opportunities created by World War I, but he offers little analysis of the antebellum period. Samuel Redman , Bone Rooms: From Scientific Racism to Human Prehistory in Museums (Cambridge: Harvard University Press, 2016), 69–73, 106–8. Warwick Anderson , Colonial Pathologies: American Tropical Medicine, Race, and Hygiene in the Philippines (Durham: Duke University Press, 2006); Mariola Espinosa , Epidemic Invasions: Yellow Fever and the Limits of Cuban Independence, 1878–1930 (Chicago: University of Chicago Press, 2009); Ann Fabian , The Skull Collectors: Race, Science, and America’s Unburied Dead (Chicago: University of Chicago, 2010); Stephen C. Kenny , ‘The Development of Medical Museums in the American South: Slave Bodies in Networks of Anatomical Exchange’, Bulletin of the History of Medicine, 87, 1 (Spring 2013), 32–62 23603528 10.1353/bhm.2013.0016; Cameron B. Strang , Frontiers of Science: Imperialism and Natural Knowledge in the Gulf South Borderlands (Williamsburg and Chapel Hill: Omohundro Institute of Early American History and Culture and the University of North Carolina Press, 2018); Khary Oronde Polk , Contagions of Empire: Scientific Racism, Sexuality, and Black Military Workers Abroad, 1898–1948 (Chapel Hill: University of North Carolina Press, 2020).

7 The scholarship on colonialism/imperialism, medicine and museums in European empires is vast. Listed here is just a starting point for scholars interested in the subject. Mark Harrison , Climates and Constitutions: Health, Race, Environment and British Imperialism in India, 1600–1850 (New York: Oxford University Press, 1999); Clifton Crais and Pamela Scully , Sara Baartman and the Hottentot Venus: A Ghost Story and a Biography (Princeton: Princeton University Press, 2009); Andrew Curran , The Anatomy of Blackness: Science and Slavery in an Age of Enlightenment (Baltimore: Johns Hopkins University Press, 2011); Michael Zeheter , Epidemics, Empire, and Environments: Cholera in Madras and Quebec City 1818–1910 (Pittsburgh: University of Pittsburgh Press, 2015); Suman Seth , Difference and Disease: Medicine, Race, and the Eighteenth-Century British Empire (New York: Cambridge University Press, 2018); James Poskett , Materials of the Mind: Phrenology, Race, and the Global History of Science (Chicago: University of Chicago Press, 2019).

8 For more on the interconnections between anti-Black racial science and expansionism in US medical education, see Willoughby, *op. cit.* (note 1), chapters 5–7.

9 William G. Rothstein, *American Medical Schools and the Practice of Medicine: A History* (New York: Oxford University Press, 1987), 34–5. On the postcolonial dynamics of eighteenth-century US medical education, see Kariann Akemi Yokota , Unbecoming British: How Revolutionary America Became a Postcolonial Nation (New York: Oxford University Press, 2011), 192–225 .

10 William G. Rothstein, *American Physicians in the Nineteenth Century: From Sect to Science* (Baltimore: Johns Hopkins University Press, 1972), 93–8. For more on the relationship between westward expansion and US medicine, see James Cassedy’s classic account, James H. Cassedy , Medicine and American Growth, 1800–1860 (Madison: University of Wisconsin Press, 1986). Likewise, Reginald Horseman’s pathbreaking work on racial thinking and manifest destiny remains instructive. Reginald Horseman , Race and Manifest Destiny: The Origins of American Racial Anglo-Saxonism (Cambridge: Harvard University Press, 1981).

11 Mary Austin Holley to J. C. Warren, 23 August 1823, John Collins Warren Papers, Massachusetts Historical Society, Boston, Mass. Rebecca Smith Lee , Mary Austin Holley: A Biography (Austin: University of Texas Press, 1962),152–4. Lee claims that Holley even sent at least one skull from Craighorn’s plantation to Warren, but her corresponding footnote makes it unclear how Lee came to this conclusion. Similarly, I have yet to find a skull that could be from this site in Harvard’s collection. Alternatively, Warren certainly traded in human remains, and the earliest known catalog of Warren’s collection was produced in 1847, so it was certainly possible that at some point Warren traded or gifted this skull to another collector but that is speculation. An example of Warren discussing trading Native American skulls can be found in my book. See Willoughby, *op. cit.* (note 1), 57.

12 Karp, *op. cit.* (note 4), 66–8, 194–8, 207–8.

13 In the introductory lecture to the 1847 session, Holmes explained to students that he undertook a large project of having images made for his lectures. He explains, ‘An experienced and faithful artist has labored constantly for several months under my immediate eye, so that I have succeeded in providing a very extensive series of illustrations …. Some of them are from works of great beauty and rarity, such as those of Albinus, Arnold, and Langenbeck, for which I am under obligations to the kindness of Professor Agassiz and of Dr. Lewis. I have ransacked every illustrated work I could find, from Vesalius to Bourgery; I have spared no man’s library, and all have been freely open to the levy *en masse* which I have not instituted’. Oliver Wendell Holmes , An Introductory Lecture, Delivered at the Massachusetts Medical College, November 3, 1847 (Boston: William D. Ticknor & Co, 1847), 33. In Holmes’s lecture notes for the 1850–1851 session, he gave three lectures on ‘races’. For the second lecture, he cited using ‘Figures from Prichard and Pickering’. In the notes for the third lecture, he described ‘figures’ as being ‘shown’. These factors lead me to conclude that at some point Holmes had enlarged images made of Pickering’s illustrated text for use in his lectures. Oliver Wendell Holmes, Holmes Lectures on Anatomy, 1850–1882, Center for the History of Medicine, Countway Library of Medicine, Harvard Medical School, Boston, MA. Charles Pickering , The Races of Man; and Their Geographical Distribution, new edition (London: H. G. Bohn, 1850). Willoughby, *op. cit.* (note 1), 100, 104–6. Charles Drake, Notes on Smith’s Anatomy Lectures, 1812–1813, Archives and Special Collections, August C. Long Health Sciences Library, Columbia University, New York, N.Y. Joseph Leidy, Envelopes 8 and 10, Notes to his Course on Anatomy at the University of Pennsylvania, undated, Medical Heritage Library, The College of Physicians of Philadelphia, Philadelphia, Pa. *Annual Announcement of the Trustees and Faculty of the Medical College of South Carolina for the Session of 1840–41* (Charleston, S.C.: Burges & James, 1840). J. L. Cabell , Syllabus of the Lectures on Physiology and Histology: Including the Outlines of Comparative Anatomy Delivered at the University of Virginia (Charlottesville, Va.: McKennie & Son, 1853). For more on Veidovi, see Fabian, *op. cit.* (note 6), chapter 4.

14 Jeffries Wyman, Journal Entry dated 9 April 1857, Jeffries Wyman Journals, Volume 1, Center for the History of Medicine, Countway Library of Medicine, Harvard Medical School, Boston, Ma.

15 For more on the importance of medical practitioners in the British Navy, see Manuel Barcia , The Yellow Demon of Fever: Fighting Disease in the Nineteenth-Century Transatlantic Slave Trade (New Haven, Conn.: Yale University Press, 2020) and Tim Lockley , Military Medicine and the Making of Race: Life and Death in the West India Regiments, 1795–1874 (Cambridge: Cambridge University Press, 2020).

16 Colonisationist was the moniker adopted by whites who wished to free enslaved people and force or encourage them to emigrate to Africa. For much of his life and political career, Abraham Lincoln, for example, was a colonisationist. Frederick Douglass, for example, ‘declared in 1852 that colonisation in any guise meant “ultimate extermination” for his people’. Douglass, in short, saw colonisation for what it was: an attempt to re-engineer the United States as a white man’s country. It should also hardly be surprising that Daniel Drake was a colonisationist. David W. Blight , Frederick Douglass: Prophet of Freedom (New York: Simon & Schuster, 2018), 238–40. Quote on 239.

17 Robert Murray draws attention to the relation between Liberia, colonisation, and medical education, as the first Black man educated in a US medical school was a Black Liberian settler, Samuel F. McGill. Robert Murray, ‘Bodies in Motion: Liberian Settlers, Medicine, and Mobility in the Atlantic World’, *Journal of the Early Republic*, 39, 4 (Winter 2019), 615–46. Francis A. Rollin , Life and Public Services of Martin R. Delany (Boston: Lee and Shepard, 1883), 68–9. Abraham R. Thompson and Joseph Fray [Managers of the Massachusetts Colonization Society] to the Medical Faculty of Harvard College, 1 November 1850 and The Medical Faculty of Harvard College to Abraham R. Thompson, 26 December 1850, Petitions and Correspondence, Re Admission of Colored Students, Center for the History of Medicine, Countway Library of Medicine, Harvard Medical School, Boston, Ma. Doris Y. Wilkinson , ‘The 1850 Harvard Medical School Dispute and the Admission of African American Student’, Harvard Library Bulletin, 3, 3 (Fall 1992), 13–27.11612967 Phillip Cash, ‘Pride, Prejudice, and Politics’, *Harvard Medical Alumni Gazette* (Dec. 1980), 22–4. For more on Liberia, see Claude A. Clegg III, The Price of Liberty: African Americans and the Making of Liberia (Chapel Hill: University of North Carolina Press, 2004).

18

Daniel Drake , John Collins Warren , and Henry Schuman , Dr. Daniel Drake’s Letter on Slavery to Dr. John C. Warren, of Boston. Reprinted from the National Intelligencer, Washington, April 3, 5 and 7, 1851, (New York: Shuman’s, 1940), 1.

19
*Ibid.*, 65–6.

20 I use the term racial scientist comparatively broadly. It refers to academics, writers and faculty that published scientific works on race and/or engaged in activities closely associated with racial science such as collecting/organising human crania into racial hierarchies. This is not to say that all medical faculty in general or even those who taught an anatomy curriculum influenced by racial science were racial scientists.

21

Jim Downs , Maladies of Empire: How Colonialism, Slavery, and War Transformed Medicine (Cambridge, Mass.: The Belknap Press of Harvard University Press, 2021), 72 .

22 For example, in discussing the potential dangers of the mass production of data on racial disparities and COVID-19, historian Merlin Chowkwanyun and political scientist Adolph L. Reed, Jr. explain that ‘data in a vacuum can give rise to biologic explanations for racial health disparities. Such explanations posit that congenital qualities unique to specific racial minorities predispose them to higher rates of a particular disease’. Merlin Chowkwanyun and Adolph L. Reed , “Racial Health Disparities and COVID-19-Caution and Context,” New England Journal of Medicine, 383, 3 (July 2020), 202 10.1056/NEJMp201291032374952.

23 For more on Bachman, monogenesis, and this consensus, see Willoughby, *op. cit.* (note 1), 31–42.

24 Karp, *op. cit.* (note 4), 164–8. To the corollary, Sharla Fett has drawn attention to how slave trade suppression provided opportunities for collecting racial data for amateur ‘ethnologists’. Sharla M. Fett , Recaptured Africans: Surviving Slave Ships, Detention, and Dislocation in the Final Years of the Slave Trade (Chapel Hill: University of North Carolina Press, 2017), 58–62 .

25 For more on Nott’s influence, see Willoughby, *op. cit.* (note 1), especially chapters 1, 2 and 7 and Reginald Horsman , Josiah Nott of Mobile: Southerner, Physician, and Racial Theorist (Baton Rouge: Louisiana State University, 1987 ). Melissa N. Stein , Measuring Manhood: Race and the Science of Masculinity, 1830–1934 (Minneapolis: University of Minnesota Press, 2015). Notably, two scholars have broken this mold of treating Nott as being a self-conscious pseudoscientist, knowingly peddling an overtly self-interested understanding of racial difference. Instead, they depict him as a complicated scientist whose views were deeply rooted in the contemporary culture of racist science and medicine. See Terence D. Keel , ‘Religion, Polygenism, and the Early Science of Human Origins’, History of the Human Sciences, 26, 2 (April 2013), 3–32 24014081 and Horsman. Josiah Nott, ‘Acclimation; or, the Comparative Influence of Climate, Endemic and Epidemic Diseases, on the Races of Men’, in Josiah Nott and George R. Gliddon (eds.), *Indigenous Races of the Earth* (Philadelphia: J.B. Lippincott & Co., 1857), 354–63, quote 1 on 354 and quote 2 on 363.

26 Nott, *ibid.*, 356–66 (quote on 356). Also quoted in Christopher D. Willoughby , ‘“His Native, Hot Country”: Racial Science and Environment in Antebellum American Medical Thought’, Journal of the History of Medicine and Allied Sciences, 72, 3 (July 2017), 341 10.1093/jhmas/jrx00328873981.

27 Nott, *ibid.*, 365–74, quote 1 on 374, quote 2 on 365.

28 Josiah Nott and George R. Gliddon (eds.), *Indigenous Races of the Earth*, (Philadelphia: J. B. Lippincott & Co., 1857), 651–6. Worth noting, many other faculty members at universities and medical schools subscribed to the book as well.

29 Samuel Henry Dickson to Josiah Nott, 16 May 1856, reprinted in Nott, *op. cit.* (note 25), 380–1, quote on 380.

30 David M. Watson, ‘An Essay on Scrofula’ (unpublished M.D. thesis, Medical College of South Carolina, 1839); Isaac Auld, ‘A Dissertation on Scrofula’ (unpublished M.D. thesis, Medical College of South Carolina, 1844), 10; Joseph Milligan, ‘An Inaugural Dissertation on Tetanus’, (unpublished M.D. thesis, Medical College of South Carolina, 1826), 2–13; Matthew Page, ‘An Inaugural Dissertation on Dysentery’ (unpublished M.D. thesis, University of Pennsylvania, 1824); James W. Boyd, ‘Intermittent Fever and Its Relation to Malaria’ (unpublished M.D. thesis, Medical College of South Carolina, 1856); 22–3; William L. Wilson, ‘An Essay on Geneanthropia’ (unpublished M.D. thesis, University of Pennsylvania, 1856), 11. For more on medical student theses as sources, see Willoughby, *op. cit.* (note 1), 12–13 and Steven Stowe , Doctoring the South: Southern Physicians and Everyday Medicine in the Mid-Nineteenth Century (Chapel Hill: University of North Carolina Press, 2004), 69–74 . Worth noting also, the Medical College of South Carolina was a regional leader in the United States South with close connections to Ivy League medical schools in the Northeast, and Charleston was often considered the centre of southern science. Lester D. Stephens , Science, Race, and Religion in the American South: John Bachman and the Charleston Circle of Naturalists, 1815–-1895 (Chapel Hill: University of North Carolina Press, 2003)16805065 and Willoughby, *op. cit.* (note 1).

31 Andrew McBryde, ‘Remarks on Some of the Most Obvious Causes of Disease’, (unpublished M.D. thesis, Medical College of South Carolina, 1837), 14–15. Similar generalisations about Africa, Africans and health can be seen in other student writing, to cite just a few here. J. Calvin Smith, ‘An Inaugural thesis on Climacterical Influences’ (unpublished M.D. thesis, Medical College of South Carolina, 1848); Tully S. Gibson, ‘An Essay on Plurality of Origin of the Races’ (unpublished M.D. thesis, University of Pennsylvania, 1855), 5; J. Frank Stinson, ‘An Essay on Intermittent Fever’ (unpublished M. D. thesis, University of Pennsylvania, 1860), 3.

32 Nott and Gliddon, *op. cit.* (note 28), 651–6. Nott and George R. Gliddon, *Types of Mankind: or, Ethnological Researches* (Philadelphia: Lippincott, Grambo, & Co., 1854), 733–8.

33 H [James Henry Hammond], “Maury on South America and Amazonia,” *Southern Quarterly Review*, (October 1853), 412–49, quote 1 on 439 and quote 2 on 444. Karp, *op. cit.* (note 4), 145–6.

34 H., op. cit. (note 33), 445–9, quote 1 on 447 and quote 2 on 449.

35 For more on the history of skull collecting and racial science in relation to anthropology, see Redman, *op. cit.* (note 6); Fabian, *op. cit.* (note 6); H. F. Augstein , James Cowles Prichard’s Anthropology: Remaking the Science of Man in Early Nineteenth Century Britain (Atlanta: Rodopi, 1999)10370858.

36 Examples of medical museums with racialised crania can be seen in the museum’s catalogs as well as from references in university circulars. William E. Horner , Catelogue of the Wistar, or Anatomical Museum of the University of Pennsylvania (Philadelphia: L. R. Bailey, 1850), 59–61 . J. B. S. Jackson , A Descriptive Catalogue of the Anatomical Museum of the Boston Society for Medical Improvement (Boston: Ticknor, 1847), 4–10 . J. B. S. Jackson , A Descriptive Catelogue of the Warren Anatomical Museum (Boston: A. Williams, 1870), 699–707 . *A Catalogue of Phrenological Specimens, Belonging to the Boston Phrenological Society*, (Boston: Printed by John Ford, 1835). R. D. Mussey, *Anatomical Cabinet*, *Belonging to R. D. Mussey* (Cincinnati: ?), 14. *Annual Announcement of the Trustees and Faculty of the Medical College of the State of South Carolina for the Session of 1840–41* (Charleston: Burges & James, 1840), 4.

37 Rouleau, *op. cit.* (note 4), 37–9.

38 By 1822, Warren was already publishing on cranial capacity and race, as can be seen in appendix H of his monograph on the comparative anatomy of the nervous system. That being said, in this period prior to the publication of Morton’s *Crania Americana* and other influential texts in support of polygenesis beginning in the late 1830s, Warren displayed a distinctly environmentalist perspective. He asserted that the ‘capacity of the improvement of the Indians for improvement are favourable’. Similarly, he claimed that African institutions combined with the tropical climate degenerated Africans, who improved remarkably in the United States. John Collins Warren, *A Comparative View of the Sensorial and Nervous Systems in Men and Animals* (Boston: Joseph W. Ingraham, 1822), 98–100, 129–43, quote 1 on 98 and quote 2 on 99. John Collins Warren Jr., ‘The Collection of the Boston Phrenological Society – a Retrospect’, *Annals of Medical History,* 3, 1 (1921), 7–9. John Collins Warren, ‘Catalogue of Preparations Deposited in the Massachusetts Medical College, November 1^st^, 1847’, Box 17, Collection Registration Records, Warren Anatomical Museum Records, 1835–2010, Countway Library, 1–13.

39 Descriptive Catalogue of the Cabinet, Volume 3, Wyman Collection, Ca. 1847–1877, Warren Anatomical Museum Records, 1835–2010 (Inclusive). 1300–4. Jackson, *A Descriptive Catalogue of the Warren Anatomical Museum*, *op. cit.* (note 36), 705–6.

40 Karp, *op. cit.* (note 4), 1–8. Cameron B. Strang , ‘Violence, Ethnicity, and Human Remains during the Second Seminole War’, The Journal of American History, 100, 4 (March 2014), 986 .

41 W. H. Prescott to John Collins Warren, 24 July 1840, John Collins Warren Papers.

42 George Gliddon to Samuel Morton, 31 March 1839, Samuel George Morton Papers, American Philosophical Society, Philadelphia, Pa. Samuel George Morton, *Catalogue of Skulls of Man and the Inferior Animals in the Collection of Samuel George Morton*, 3^rd^ edn. (Philadelphia: Merrihew & Thompson, 1849), 91, 95–6, 98–100. Daniel R. Headrick , The Tools of Empire: Technology and European Imperialism in the Nineteenth Century (New York: Oxford University Press, 1981), 150–6.

Heather Streets-Salter and Trevor R. Getz . Empires and Colonies in the Modern World, 2nd edn. (New York: Oxford University Press, 2016), 248–50. Other historians have discussed how empires allowed for the emergence of modern racial science. See Efram Sera-Shriar, ‘Ethnology in the Metropole: Robert Knox , Robert Gordon Latham and Local Sites of Observational Training’, Studies in the History and Philosophy of Biological and Biomedical Sciences, 42, 4 (December 2011), 486–9610.1016/j.shpsc.2011.07.00422035722 and Helen MacDonald , Human Remains: Dissection and its Histories (New Haven: Yale University Press, 2005).

43
*A Catalogue of Phrenological Specimens, Belonging to the Boston Phrenological Society*, (Boston: Printed by John Ford, 1835), Box 10, Folder 1, Collection Registration Records, Warren Anatomical Museum Records, 1835–2010, 29, 39. Object 373a is a handwritten addition to the text. Descriptive Catalogue of the Cabinet, Volume 3, Wyman Collection, Warren Anatomical Museum Records, 1835–2010, Object 747. ‘Descriptive Catalogue of the Cabinet, Volume II, Wyman Collection, Ca. 1837–1847’, Folder 11, Box 10, Collection Registration Records, Warren Anatomical Museum Records, 1835–2010, 284–5.

44
*The Congregationalist*, Boston, Ma., 15 January 1861. Jackson, *A Descriptive Catalogue of the Warren Anatomical Museum*, *op. cit.* (note 36), 702.

45 For a detailed description of my method for recreating the organisational system of the Warren Museum, see Willoughby, *op. cit.* (note 1), Chapter 3, Note 52. Worth noting, these skull collections predate the popularity of evolutionary theories of human difference among scientists in the United States. John Collins Warren, for example, expressly disavowed Larmarckian evolution. John Collins Warren, Entries dated 24 August and 26 August 1838 entries, Volume 72, John Collins Warren Papers.

46 Washington was originally part of the Oregon Territory established in 1848 before being separated in 1853.

47 For example, in 1837, Philadelphia physician and racial scientist Samuel G. Morton asked Warren to trade him the skulls of ‘two flat headed Indians’. Warren refused, because ‘they [were] so different, so peculiar, and so useful’. John Collins Warren to Samuel Morton, 3 February 1837, Samuel George Morton Papers, American Philosophical Society, Philadelphia, Pa. Samuel George Morton to J. C. Warren, [month & day unclear]1837, John Collins Warren Papers. Also quoted in, Willoughby, *op. cit.* (note 1), 57. It was Warren’s theory that the first Native Americans were likely of South Asian descent and founded the Native American civilisations of Peru, Central America and the Mississippi River Valley. These were advanced civilisations with impressive architecture, as Warren saw it. In contrast, the Native Americans that whites had displaced and warred with were a second, racially distinct group from the first, who ultimately defeated and interbred with the Native Americans that Warren perceived as civilisation builders. In short, whites, as Warren saw it, displaced inferior invaders not the creators of great civilisations like the Peruvians. Undated entry between 13 July and 14 September, 1837, Volume 70: Journal, 13 July-–14 September 1837, John Collins Warren Papers.

48 While the exact number of Native American remains stolen and displayed is difficult to quantify precisely, through the Native American Graves Protections and Repatriation Act (NAGPRA) passed into law in November 1990, we can make sound estimates for those that remained in the United States. As of 2020, 67 000 sets of Native American remains had been repatriated, but it is believed that 127 000 sets of remains still reside on museum shelves. Stephen E. Nash and Chip Colwell , ‘NAGPRA at 30: The Effects of Repatriation’, Annual Review of Anthropology, 49, 1 (2020), 225 . Wendi A. Lindquist , ‘Stealing from the Dead: Scientists, Settlers, and Indian Burial Sites in Early-Nineteenth-Century Oregon’, Oregon Historical Quarterly, 115, 3 (Fall 2014), 324–43.

49 “Descriptive Catalogue of the Museum of the Mass. Medical College,” August 1850, 420. “Second Catalogue,” 1861, 34. Warren Anatomical Museum Records, 1835–2010 (inclusive), 1971–1991 (bulk). For more on anatomical preservation techniques, see William Horner, *The United States Dissector, or Lessons in Practical Anatomy*, 4^th^ edn. (Philadelphia: Blanchard & Lea, 1846). Harvard also purchased the man’s sternum in four pieces, a ‘dark brown, congenital mark, with hair upon it; from a Flat-head Indian’, and ‘mesenteric glands, extensively tubercular, and lacteals filled with the same deposit. From a Flat-head Indian’. J. B. S. Jackson, *op. cit.* (note 36), 5, 50, 378 and 703. Harvard actually has receipts for both the man’s exhumation and the casting of his remains. Receipt from L. L. Tarbell Sr., Funeral Undertaker, 9 May 1860 and Receipt from Pietro Garibaldi, 12 May 1860, Warren Anatomical Museum Bills, Records of the Warren Anatomical Museum, 1828–1892.

50 For discussion of the origins of Tulane’s possession of the mummies, which they still have, see Nott and Gliddon, *op. cit.* (note 32), 132, 428–9. Louis J. Jordan, *Hand-Book & Descriptive Catalogue of the Pacific Museum of Anatomy and Natural Science* (San Francisco: ?, 1865?), 44–55, quote 1 on 44, quote 2 on 50, quote 3 on 54, quote 4 on 55.

51 Mussey, *op. cit.* (note 36), 5–14, quote on 14. In my brief discussion of Mussey’s collection of *Masters of Health*, I referred to his collection of ‘human crania’ as having seventeen skulls, but did not go into detail about how Mussey’s collection included two ‘deformed crania’.

52 Mussey, *op. cit.* (note 36), 14. For more on the interconnected construction of disability and race, see Barclay, *op. cit.* (note 3).

53 Mussey, *op. cit.* (note 36), 14–19, quote 1 on 17 and quote 2 on 19.

54 For more pharmaceutical testing in the global south, see Sonia Shah , The Body Hunters: Testing New Drugs on the World’s Poorest Patients (New York: The New Press, 2006).

